# Catatonia: A Common Cause of Late Regression in Autism

**DOI:** 10.3389/fpsyt.2021.674009

**Published:** 2021-10-28

**Authors:** Mohammad Ghaziuddin

**Affiliations:** Department of Psychiatry, University of Michigan, Ann Arbor, MI, United States

**Keywords:** catatonia, autism, adolescence, late regression, comorbidity

## Abstract

Autism spectrum disorder (ASD) is a neurodevelopmental disorder characterized by social communication deficits and restricted interests and behaviors which begin very early in life. In about a quarter of cases, the symptoms emerge about 18–24 months after a period of normal development, a phenomenon commonly described as early regression. However, marked functional decline can also occur in persons with autism after a relatively stable childhood. As opposed to early regression, which occurs in normally developing children, late regression occurs typically in adolescents with an established diagnosis of autism. Apart from their occasional mention in the literature, these individuals have not been examined systematically. This Brief Report describes the presentation, comorbidity and short-term outcome of 20 persons with ASD who developed late regression. The mean age of onset of regression was 13 years. One of the earliest symptoms was an increase in obsessive slowing and compulsive rituals. Other symptoms included motor abnormalities, aggression and mood disturbance. The most common comorbid disorder was catatonia occurring in 17 patients. Despite treatment with several modalities, the outcome was often suboptimal. These findings suggest that catatonia is a common cause of late regression in persons with autism. Clinical and research implications are discussed.

## Introduction

Autism spectrum disorder (ASD) is a severe handicapping disorder of early childhood characterized by social communication deficits and restricted interests. About a quarter of children with autism are believed to regress during early childhood, usually between 18–24 months. However, some patients experience severe functional decline after a relatively stable childhood usually during adolescence or early adulthood, a phenomenon that can be called late regression. As opposed to early regression, this process occurs in persons who have already been diagnosed with autism. It differs from commonly occurring behavioral problems of adolescence by its severity and pervasiveness, and by its poor response to treatment. Although most experienced child psychiatrists have come across persons with autism who begin to deteriorate significantly during adolescence after a stable childhood, little attention has been paid to them with some exceptions. Thus, Rutter reported that seven out of 64 (10.9%) patients showed a progressive deterioration during adolescence which began with “a loss of language skills associated with inertia and decreasing activity followed by a general intellectual decline” (1). In three of these patients, deterioration was accompanied by “epileptic fits and in a fourth with paralysis of the legs.” Six patients were admitted to a long stay hospital and never discharged; however, one patient, who had severe comorbid obsessive-compulsive disorder, was hospitalized for 6 months and made complete recovery. Billstedt and colleagues reported higher estimates of this group of patients. Out of a total sample of 120, 24 (20%) deteriorated during adolescence and in 12 out of these, the “deterioration appeared to be permanent” (2). Kobayashi and colleagues surveyed 201 adults with autism (mean age 21.5 years) who had participated in educational and health services. On the basis of questionnaires and direct examination, they reported that one third of their patients deteriorated after adolescence (3). Because of decreasing number of long-stay psychiatric beds, and possibly because of increasing awareness of the psychiatric comorbidity of autism across the life span, patients who meet the definition of late regression are being increasingly referred to emergency rooms and acute psychiatric units. This Brief Report attempts to characterize the presentation and short-term outcome of 20 adolescents with ASD who developed late regression, with particular reference to the comorbidity with catatonia.

## Methods

From a series of 13 to 17 year-old adolescents with ASD (4) referred to the psychiatry department of an academic medical center, cases with late regression were identified. For the purpose of this study, late regression was defined as a significant decline in social, communication and other areas of functioning, occurring in a relatively well-functioning adolescent with autism. Degree of severity of the deterioration was indexed by an urgent referral to the outpatient clinic, the emergency room, or admission to the inpatient unit. Patients with chronic behavioral problems *without* significant qualitative and quantitative deterioration during adolescence were excluded.

Data were abstracted from a checklist completed by parents and caregivers. This checklist consisted of 30 psychiatric symptoms rated on a scale of 0 to 2 (not present, sometimes present and often present), and details about family psychiatric history, life events, school details, medications used, and past psychiatric history. In addition, the following rating scales were collected: Child Behavior Checklist (5); Reynold's Adolescent Depression Scale-2 (6); Multidimensional Anxiety Scale for Children (7); Conner's Rating Scales-Revised (8); Bush-Francis Scale for catatonia (9); and the Social Communication Questionnaire for autism (10). The diagnosis of ASD and of comorbid psychiatric disorders was based on the DSM 5 and made by an experienced child psychiatrist after repeated examination of the patient on at least three occasions. Structured interviews and observation scales for diagnosing autism were administered only if the diagnosis of autism had previously not been made and if the patient was clinically stable. Categorization of intellectual disability was based on the IQ and/or details of adaptive functioning gathered from history and records.

Laboratory investigations consisted of complete blood picture, metabolic panel, thyroid function tests, urinalysis and drug screen. EEG, MRI, NMDA receptor antibodies, antistreptolysin O titer, urine organic acids, and a chromosomal microarray were also performed when indicated. Duration of the follow-up ranged from 1 to 3 years. Outcome was based on Lotter's method and classified as “good” (normal or near normal social life and satisfactory functioning at school or work); “fair” (some social and educational progress, in spite of significant, even marked, abnormalities in behavior or interpersonal relationships); and “poor” (severe handicap with no independent social progress) (11). The study was approved by the institutional IRB.

## Results

Out of 26 patients eligible to participate in the study, six were excluded leaving a sample of 20 patients (12 males, 8 females; age 13–17; mean 15.5 years). Based on their intellectual ability and adaptive functioning, eight were classified with high functioning autism (HFA) and 12 with low functioning autism (LFA). All the patients were treated on the inpatient during the acute phase and followed up in the outpatient clinic.

The mean age of onset of late regression was 13 years (range 10–15 years). Presenting symptoms consisted of an increase in obsessive slowing and ritualistic behaviors; severe aggressive behavior either of a recent onset or marked worsening of pre-existing behavior; intense mood symptoms; complaints of auditory or visual hallucinations; eating problems with excessive or minimal food intake; marked changes in the speech output including muteness; and a combination of these symptoms. Based on rating scales and direct observation, the most common comorbid diagnosis was catatonia (*n* = 17, 85%) followed by disruptive behavior disorder (*n* = 10, 50%); mood disorder (*n* = 10, 50%) and psychotic disorder (*n* = 7, 35%). Several patients had multiple diagnoses. Fifteen (75%) had a family history of psychiatric illness in the first-degree relatives. Three (15%) had a history of early regression before the age of 3–4 years. Seven patients had chromosomal abnormalities including two with Down syndrome (Trisomy 21), one with Di George Syndrome (22qdel Syndrome) and one with 15 q duplication syndrome (see Table 1). One patient had cerebellar ectopia and another had an arachnoid cyst. All the patients had been exposed to psychotropic medications at the time of referral or in the past.

**Table 1:** Demographic characteristics of the sample.

Number	20
Male: Female	12:8
Mean Age (range)	15.55 (13–17)
HFA: LFA	8:12
Mean age of onset of adolescent-regression (range)	13.00 (12–15)
Family history of psychiatric illness	15
History of early regression	3
History of infection/surgical procedures	2
Chromosome abnormalities	Trisomy 21 (Down syndrome 2 cases); One each with Charge syndrome (22q del); 15q dup syndrome; Xq28 dup; 9p24.2 del; 3q13.13.
MRI findings	Cerebellar ectopia 1; Arachnoid cyst 1
Catatonia	17
Eating Disorder	4
Outcome	Fair (12); Poor (6); Good (2)

Treatment occurred in both inpatient and outpatient settings and consisted of a combination of medications and therapy. In general, those with normal intelligence and reasonably good verbal skills received supportive and cognitive behavioral therapy while those with intellectual disability and behavioral problems received medications with behavioral therapy. Ten patients received electroconvulsive therapy (ECT).

At follow-up after 1–3 years, only two patients were rated as having a good outcome (described as having reached near-normal premorbid level of functioning). 12 patients had a “fair” outcome because of “some social and educational progress in spite of significant abnormalities in behavior or interpersonal relationships” and six had a “poor” outcome (defined as severe handicap with no independent social progress). Thus, only two patients (10%) almost reached their premorbid level of functioning.

## Discussion

This preliminary study describes a group of children with ASD who developed severe decline in multiple areas of functioning during adolescence. Because of its age of onset, severity, and generally poor outcome, this period may be labeled as late regression of autism.

Obsessive slowing and increased ritualistic behaviors were the most common presenting features along with mood and behavioral changes. Parents often complained of such behaviors as “freezing moments” and “getting stuck in the doorways.” Other symptoms were new-onset aggression with pressured speech and overactivity resembling hypomania, and reports of “hearing voices and seeing visions.” However, these “hallucinations” were often ill-defined and usually accompanied by mood changes.

The most common comorbid disorder was catatonia occurring in 17 (85%) out of the 20 patients. Nine were males, and seven females, with a mean age of 14.5 years, which is a year less than that of the entire sample. 12 of the catatonic patients were low functioning suggesting that catatonia and intellectual disability may increase the risk of late regression in autism. Despite the initial diagnosis of psychosis in seven patients in the catatonia group, none met the criteria of schizophrenia. A clear history of abnormal eating going back to early childhood was present in three patients with catatonia; one had a history of hyperphagia, one of rumination disorder, and another of ARFID (4). Three patients with catatonia had a history of epilepsy but none had active seizures at the time of the study. Similarly, infection was suspected in two cases but could not be confirmed on repeated interviews and examinations. Eight patients with catatonia received ECT and nine were treated with medications. At 1–3 year's follow-up, two patients with catatonia received a rating of “good,” 12 of “fair” and 5 of “poor.” The high number of patients with catatonia among adolescents with late regression underscores the need for screening for this condition in persons with developmental disorders including those with autism (12). While catatonia was the most common comorbid diagnosis in the sample, it is unclear to what extent it was a causative or a contributory factor in the etiology of late regression. In addition, it is important that 15% of the sample did not have catatonia suggesting that catatonia is not the only disorder that can be associated with or be the cause of late regression in autism. In the present sample, the three patients who had regression without catatonia had severe mood disorders including two with obsessive compulsive disorder. A larger study with a proportional number of patients without catatonia will help clarify some of these points.

A family history of psychiatric illness was common. In 15 patients, first degree relatives were affected, usually with mood disorders. The yield of biological investigations, such as EEG and MRI, was low. One patient had cerebellar ectopia and another showed an arachnoid cyst on the MRI. In all, seven patients had positive findings on Chromosomal Microarray Array (CMA). Two patients had Down syndrome (trisomy 21) and one each had 22q deletion (Di George Syndrome), and 15q duplication syndrome. Of the remaining three patients with findings of uncertain significance, one each had 3q13.13 intragenic deletion, 9p24.2 deletion, and X28q duplication. All the patients with chromosome abnormalities had catatonia. Although systematic studies of the genetic abnormalities in patients with autism undergoing late regression have not been done our findings are consistent with those of a recent study by Verhoeven et al. (13). In this study of Phelan McDermid syndrome, characterized by deletion of the SHANK3 gene located on 22q13.33, the authors found that out of 24 adult patients, four developed regression during early adulthood and two of these had autism.

Treatment consisted of multiple medication trials and behavioral therapy. None of the patients were medication naïve. Out of the three patients without catatonia who had severe comorbid mood disorders, one received medications and the remaining two received ECT. In all, ten patients received ECT and out of these, eight had catatonia. As part of the standard treatment for cases with catatonia, antipsychotic medications were gradually reduced when appropriate and a trial of benzodiazepines (Lorazepam) administered in increasing doses. ECT was considered under the following circumstances: If benzodiazepines were not effective in treating catatonic symptoms after a reasonable period; if there were contraindications to their use; or if the patient's overall condition demanded it. ECT was administered after an independent evaluation by three experienced child psychiatrists and obtaining an informed consent from the guardians. Pre-medications used were succinylcholine (Brevital, as a muscle relaxant) and romazicon (Flumazenil, if the patient was receiving benzodiazepines). All the patients were treated by bilateral electrode placement. The usual electric charge was 20 to 40 mC; further increase in the charge was individualized according to the adequacy of the seizure (average duration about 30 s). The range of the number of ECT ranged from 27 to 134 over a 1year period.

On the whole, the outcome was rated as “fair” in 12 patients, “poor” in six patients, and “good” in only two patients. The two patients who were rated as having achieved a “good” outcome were males with high functioning autism, one with catatonia and the other with obsessive compulsive disorder. Out of the ten from the entire sample who received ECT, seven had a fair outcome and three had a poor outcome. Eight of the 17 patients with catatonia received ECT, which is generally regarded as the treatment of choice in catatonia (14). Out of these eight patients, the outcome was rated as “fair” in six and as “poor” in two patients. However, outcome is influenced not only by the type of treatment but also by other factors, such as, the severity of symptoms, the criteria used for measuring outcome, and the duration of follow-up of the study. In this short-term study, “fair” outcome was defined as “some social and educational progress in spite of significant abnormalities in behavior or interpersonal relationships”; and a “poor” outcome was defined as severe handicap with no independent social progress. Therefore, most of the patients with catatonia did show some degree of social and educational progress, although an overwhelming number of patients did not reach their premorbid level of functioning prior. A long-term study based on a large sample is needed to shed light on the treatment of catatonia in the context of late regression in persons with autism.

While the findings are important, they have to be interpreted with caution because of their potential limitations. First, the sample consisted of a heterogeneous group of patients referred to a tertiary referral center. This occurred because severely impaired patients meeting the inclusion criteria are treated either in hospitals or in residential treatment centers. Second, it may be argued that some of the patients may not have had autism but other neurodevelopmental disorders. For example, seven had chromosomal abnormalities and 17 out of the 20 had catatonia. However, autism can occur *with* a variety of other medical disorders and genetic syndromes associated with intellectual disability (15). This is illustrated by the two patients with trisomy 21 Down syndrome. The first patient was a 15 year old male who was first referred to his pediatrician at the age of 4 years because of increasing behavioral problems consisting of hyperactivity; aggression toward his sister; a tendency to indulge in repetitive behaviors such as throwing objects, spinning toys, clearing surfaces of furniture etc. The parents reported that it was difficult for him to play alone or with others. He did not respond to his name being called and his vocabulary consisted of a few words which he seldom used appropriately. By that time, he had already been diagnosed with autism by the department of physical medicine, but his school had insisted on a second opinion. When referred to our clinic at the age of 5 years for a second opinion, he displayed the followed clinical features: extreme hyperactivity requiring 1:1 supervision at school; aggressive outbursts when things did not go his way; impaired social interaction and communication including poor eye contact; and lack of appropriate play with other children. At the age of seven years, he was evaluated again in the child psychiatry clinic. By this time, he was receiving educational services for children with autism. Some of the behaviors during the clinic visit were described as follows: “Poor eye contact, extreme hyperactivity, tics and stereotypies including arm movements. Play was repetitive and perseverative with a rubber frog that he persisted in throwing up in the air and catching. He lined some cards that he brought with him.” A diagnosis of autism was, therefore, confirmed along with that of intellectual disability and trisomy 21. The second patient with Down syndrome, a 13 year old female, was first seen by the department of pediatrics in our hospital system at the age of seven years. At that time, she was already receiving speech, occupational and physical therapy. The examining pediatrician noted severe sensory problems and behavioral outbursts. Parents reported that the following baseline-behaviors: social withdrawal; preference to spend time alone; repeating of phrases out of context (eg., “Go Robin, Go Robin”); stereotypies or quick movements of her fingers; rigidity regarding her schedules etc. On the basis of this history and the examination on several occasions by clinicians (pediatricians and child psychiatrists) and reports from collateral sources such as school personnel, a diagnosis of autism with Down syndrome was confirmed.

Third, it may be argued that despite emerging interest in the topic, the diagnosis of late regression in autism remains controversial. To address this concern, regression was defined as a period of severe pervasive functional decline occurring in a relatively stable patient with a history of autism. To differentiate it from symptoms of other conditions that can begin during early childhood, the functional decline of late regression had to emerge after a relatively symptom-free compensated period; had to be extreme in severity; last for at least 6 months; and affect multiple areas of functioning such as communication, mobility and self-care. Thus, we excluded patients whose symptoms were chronic. To help define the onset, we obtained a detailed history incorporating anchor dates, collateral information, and repeated interviews. Fourth, biological measures could not be obtained in all cases because of clinical reasons and limitation of resources. Finally, a control group was not included in the study because the main purpose of this preliminary study was to initially define and characterize the presentation of late regression in persons with autism and generate testable hypotheses. Systematic studies based on large numbers, drawn from multiple settings, are required to identify the etiology, and the risk and vulnerability factors, including its comorbidity with catatonia, to facilitate its early diagnosis and treatment.

## Data Availability Statement

The raw data supporting the conclusions of this article will be made available by the authors.

## Ethics Statement

The studies involving human participants were reviewed and approved by University of Michigan Medical IRB. Written informed consent from the participants' legal guardian/next of kin was not required to participate in this study in accordance with the national legislation and the institutional requirements.

## Author Contributions

The author is responsible for the design, data extraction, and all aspects of the preparation of the manuscript.

## Conflict of Interest

The author declares that the research was conducted in the absence of any commercial or financial relationships that could be construed as a potential conflict of interest.

## Publisher's Note

All claims expressed in this article are solely those of the authors and do not necessarily represent those of their affiliated organizations, or those of the publisher, the editors and the reviewers. Any product that may be evaluated in this article, or claim that may be made by its manufacturer, is not guaranteed or endorsed by the publisher.
